# Traditional Applications of Tannin Rich Extracts Supported by Scientific Data: Chemical Composition, Bioavailability and Bioaccessibility

**DOI:** 10.3390/foods10020251

**Published:** 2021-01-26

**Authors:** Maria Fraga-Corral, Paz Otero, Lucia Cassani, Javier Echave, Paula Garcia-Oliveira, Maria Carpena, Franklin Chamorro, Catarina Lourenço-Lopes, Miguel A. Prieto, Jesus Simal-Gandara

**Affiliations:** 1 Nutrition and Bromatology Group, Analytical and Food Chemistry Department, Faculty of Food Science and Technology, Ourense Campus, University of Vigo, 32004 Ourense, Spain; mfraga@uvigo.es (M.F.-C.); pazoterofuertes@gmail.com (P.O.); lucia_cassani@hotmail.com (L.C.); javier.echave@uvigo.es (J.E.); paula.garcia.oliveira@uvigo.es (P.G.-O.); maria.carpena.rodriguez@uvigo.es (M.C.); chamorro1984@gmail.com (F.C.); clopes@uvigo.es (C.L.-L.); 2 Centro de Investigação de Montanha (CIMO), Campus de Santa Apolonia, Instituto Politécnico de Bragança, 5300-253 Bragança, Portugal; 3 Department of Pharmacology, Pharmacy and Pharmaceutical Technology, Faculty of Veterinary, University of Santiago of Compostela, 27002 Lugo, Spain; 4 Research Group of Food Engineering, Faculty of Engineering, National University of Mar del Plata, Mar del Plata RA7600, Argentina

**Keywords:** tannins, pharmacological, medicinal, veterinary, nutritional, traditional application, traditional use, human and animal health

## Abstract

Tannins are polyphenolic compounds historically utilized in textile and adhesive industries, but also in traditional human and animal medicines or foodstuffs. Since 20th-century, advances in analytical chemistry have allowed disclosure of the chemical nature of these molecules. The chemical profile of extracts obtained from previously selected species was investigated to try to establish a bridge between traditional background and scientific data. The study of the chemical composition of these extracts has permitted us to correlate the presence of tannins and other related molecules with the effectiveness of their apparent uses. The revision of traditional knowledge paired with scientific evidence may provide a supporting background on their use and the basis for developing innovative pharmacology and food applications based on formulations using natural sources of tannins. This traditional-scientific approach can result useful due to the raising consumers’ demand for natural products in markets, to which tannin-rich extracts may pose an attractive alternative. Therefore, it is of interest to back traditional applications with accurate data while meeting consumer’s acceptance. In this review, several species known to contain high amounts of tannins have been selected as a starting point to establish a correlation between their alleged traditional use, tannins content and composition and potential bioaccessibility.

## 1. Introduction

Tannins have been used throughout history for their pharmacological properties as part of plants and herbs in traditional medicine. Also, they have been extensively used since the 18th Century by leather manufacturers to improve leather resistance in the dyeing or tanning process, as they can precipitate gelatin adhered to animal skin and provide a brownish color. Hence, the name of this group of phytochemicals [1]. Tannins are a heterogeneous group of polyphenols, secondary metabolites in plants synthesized in response to biotic and abiotic stress inducers. The phenolic rings and hydroxyl groups present in their chemical structures confer them antioxidant and protein-binding properties, as they have a wide range of molecular weight (500–20,000 Da) that it also showed in their broad structure diversity [2]. By their nature and abundance of hydroxyl radicals, tannins are highly hydrophilic molecules, soluble in aqueous solvents as well as exhibiting a high tendency to stably bond with proteins and carbohydrates [3]. This feature is common to all tannins, yet it seems that their link with polysaccharides lowers the probability of bonding and interacting with proteins [4]. They also share other properties, as the precipitation of colored complexes with iron salts or oxidation by potassium permanganate in alkaline media. Tannins are ubiquitously present in barks, seeds or fruit peels of many vegetable species, but also in brown algae [5]. Although several categorizations have been made on tannins regarding their molecular weight, properties and source, tannins are widely accepted to be classified under their functional units. As such, hydrolyzable tannins (HT), proanthocyanidins or condensed tannins (CT) and complex tannins (CoT) can be found on terrestrial plants, while phlorotannins (PT) have only been reported in brown macroalgae [4]. Among tannin diversity, the most abundant are terrestrial tannins, of which CT are generally most common. Even though their concentration and class differ in the different fractions of plants, tannins seem to have similar properties such as antioxidant, antimicrobial or predator-deterrent (i.e., against helminths or herbivores) [6].

Regarding tannins structure, galloyl units are the bricks that form HT, but depending on their chemical unions and radicals, they may be differentiated as gallotannins (GT) or ellagitannins (ET), relying upon the presence of gallic acid (GA) or ellagic acid (EA) subunits on degradation [7]. As such, they are synthesized from the shikimate pathway [2]. In general terms, GT are polymers of galloyl coupled with polyol, catechin or triterpenoid units, frequently found in the form of pentagalloyl glucose (PGG). The complexity of HT grows as more galloyl units are coupled through meta- or para-depside bonding, forming a chained structure of ester (oxidative) bonds [8]. ET are mainly galloyl units organized through C–C bonds such as in hexahydroxydiphenol (HHDP), HHDP-esters or nonahydroxytriphenoyl (NHTP) esters subunits [6]. The hydrolyzation of the HHDP subunit prompts EA subunits. The hydrolyzable label of HT indicates its low resistance to be hydrolyzed by high temperatures, acids, bases and specific enzymes such as tannase, commonly resulting in pyrogallol or GA products [9]. However, many ET are much more resistant to hydrolyzation because of the additional C–C bonding of their polyphenolic residue with the polyol unit [10].

The CT structure is regularly built upon catechins or epicatechins, the most common being (2,3-trans)-(±)-catechin and (2,3-cis)-(±)-epicatechin, which are flavan-3-ols moieties [11]. They are thus originated on the flavonol pathway [12]. The polymerization of CT is usually formed by bonding other catechins through C4-C8 bonds, but C4-C6 bonds may also be created, albeit less frequently [5]. The position of hydroxyl groups gives away a variation on their hydroxylation pattern in the A and B ring of the flavanol-3-ol unit, which in turn provides the classification of several groups of CT such as procyanidins (3,5,7,3′,4′–OH), prodelphidins (3,5,7,3′,4′,5′–OH), propelargonidins (3,5,7,4′–OH), profisetinidins (3,7,3′,4′–OH), prorobinetinidins (3,7,3′,4′,5′–OH) or proteracacinidins (3,7,8,4′–OH) among others. Among these groups, procyanidins are the most abundant in nature, which can be sorted on the linkage between flavanyl units in A (double), B or C (single) class [13,14].

On the other hand, CoT are tannins of high molecular weight resulting from the bonding of flavan-3-ols with either GT or ET via a C–C bond. Some examples of CoT are acutissimins A and B, which can be isolated from *Quercus* sp. and *Castanea sativa* or camelliatannin A from *Camelia japonica* [3].

**Figure 1:**
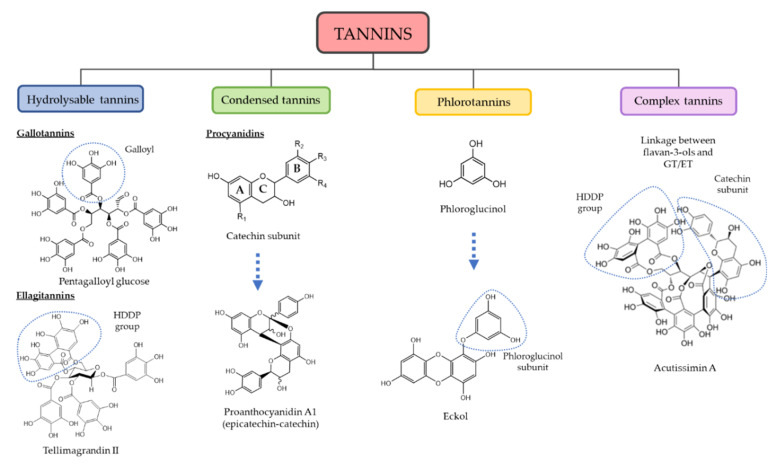
Classification and general representative structures of tannins. Functional groups are circled. Rings in catechin molecule are labeled as A, B and C. R = radical, H, OH; GT = gallotannins; ET = ellagitannins; HHDP = hexahydroxydiphenol.

PT are common tannins present in algae and constituted upon molecules of phloroglucinol (PG, aromatic ring with 1,3,5 hydroxyl groups) that polymerize with ease between C1-C3. They are grouped into three distinctive classes based on the coupling between subunits: fucols (C–C), phloroetols (C-O-C) and fucophloroteols (C–C and C-O-C). Increasing complexity in their structure is correlated to a higher presence of PG subunits (3 to 7 subunits) [15]. As well as terrestrial tannins, PT exert, in some cases, antimicrobial protection while their potent antioxidant properties confer protection against UV-A and UV-B radiation [6]. A general perspective of tannin classification attending to their structure is presented in Figure 1.

Generally, tannins are accumulated in vegetable cells in a special vacuole of recent discovery called tannosome, from which they are secreted to tissues. The inclusion of tannins in this vacuole avoids a disruptive binding of tannins with metabolic proteins or polysaccharides [13]. In the case of ET, it is worth noting that they show much lower protein binding activity at low/neutral pH, in contrast with the rest of the tannin groups [6]. Yet, this is not always the case, as they may also be embedded in cell walls. This is most prominent in the case of PT since they are integrated into the cell wall and bound to algal polysaccharides like alginate, laminarin or fucoidan [16]. Correlating to their biosynthesis pathways, different classes of tannins do not tend to accumulate simultaneously in the same tissue, as their concentrations may change with environmental conditions (i.e., seasonal changes) and plant tissue structural characteristics [17].

Tannins have rather undesirable organoleptic properties, as they are bitter and give a brownish color to foods. Nevertheless, they show remarkable antioxidant properties that justify their use as food additives to improve food-shelf life and safety, an issue that has made several tannins undergo trials for their legal approval as such additives. Furthermore, their precipitation properties are accounted for their decades-long use as clarification agents in the beverage industry (i.e., in beer, juices and wines) [18]. For instance, in the case of wines, ET from oak or chestnut are transferred to the wine during barrel aging, which is an appreciated feature in aged wines. As another example, tannic acid, a common GT found in many species, is approved as a flavoring agent in the EU [19]. However, it has also been reported alongside many other tannins to provide further oxidative and antimicrobial protection when added to foods [4]. In the same sense, several in vitro, in vivo and clinical studies researching the bioactive properties of tannins have been developed throughout the years [20]. Thus, it is evidenced by their polyvalent potential, whether as additives, nutraceuticals or pharmaceutics. The mentioned findings are paired with increased consumer demand for natural products, with a preference to avoid or replace synthetic compounds in food, for example [21]. Furthermore, the feasibility of tannin extraction and acquisition proves that it is affordable and may be carried out with little difficulties even from by-products of the agri-food industry, such as barks, leaves, peels or seeds that are not exploitable for other uses, since these fractions are accounted for the highest tannin concentrations. Among these plant tissues, some remarkable examples involve peels and/or seeds from several fruits (i.e., grape, pomegranate), citrus, nuts (i.e., chestnut, walnut), herbs (i.e., tea, basil, cinnamon), legumes and barks of trees (i.e., *Acacia* spp., *Castanea* spp., *Quercus* spp.) [17,22,23,24]. Some common sources of currently commercialized tannin extracts are trees such as chestnut (*C. sativa*) for HT and quebracho (*Schinopsis balansae* & *Schinopsis lorentzii*) for CT [25]. In the case of these woody trees, tannin content in barks may be as high as 38% of dry matter in quebracho or as much as 16% in chestnut, and their use in traditional medicine is well recorded. Other usual sources of tannins are black wattle (*Acacia mearnsii*) for CT or valonea (*Quercus macrolepsis*) and tara (*Tara spinosa*) for ET and GT, respectively [26]. Furthermore, as stated, brown algae (i.e., Arame, *Eisenia bicyclis*, *Sargassum* sp.) are also considered an adequate source of tannins, as well as other bioactive compounds, as they are easily harvested and currently underutilized while being the focus of research on bioactive compounds in recent years [27].

Taking into account the mentioned properties of tannins, it may be possible to explain the effectiveness of tannin-rich medicinal plants used in traditional medicine while these medicinal properties are also related to the synergy of tannins with other bioactive polyphenols present in these plants [28]. The recorded medicinal use of tannin extracts and tannin-rich plants will be addressed together with the study of the main chemical profile of the mentioned tannin-rich plants. Therefore, the main objective of this review is to relate the traditional knowledge gained after centuries of application of traditional medicine with scientific data that may point to the target molecules.

## 2. Traditional Applications of Rich-Tannins Plants

In the following sections and in Table 1, some examples of the traditional uses of plants and other sources of tannins will be explained. The selection of these species has been made according to their well-known content in tannins, their extensive recorded traditional applications (paying special attention to those orally administrated), their reported bioactivities and the availability of quantitative and qualitative studies that determined their chemical profile and their high levels of tannins.

Among the species of the genus *Acacia*, *A. nilotica* is the most relevant from a medicinal point of view. Different parts of the plant have been used for very diverse affections. Even though all tissues have been described to possess activity, leaves, pods and bark present more healing properties. In general terms, this species has been described to treat gastrointestinal disorders or diseases (diarrhea, congestion, anthelmintic, diuretic, emetic, for burning sensation and it is also considered as nutritive), respiratory affections (pharyngitis, bronchitis, cough, cold, expectorant and for sore throat), skin issues (eczema, ulcers, leukoderma, wounds), variable inflammatory processes (toothache, conjunctivitis, menstrual pain, hemorrhoids, smallpox, biliousness) or diabetes. Its sedative and narcotic properties were applied for nervous system disorders, Alzheimer’s disease and its antimicrobial capacity was exploited as a remedy for dysentery, leprosy, tuberculosis or even malaria. It also possesses aphrodisiac properties, it can be used for treating spermatorrhoea and sexually transmitted diseases, but it was also claimed to possess chemo-preventive and antimutagenic activity [29,30]. The properties recognized for major tannins in *A. nilotica* include antioxidant, anti-inflammatory, anti-nociceptive, and antipyretic activities [31,32]. Another plant belonging to this genus with recognized properties is *A. tortilis* sap, whose seeds and bark recovered have been used for gastrointestinal ailments (stomachache, mild diarrhea or indigestion), eye conditions (treat white stains in the cornea or used in incipient cases of eye entropy), respiratory issues, as antipyretic, for jaundice, malaria, as wound healing and injuries disinfectant, as liver detoxifying and for bone strengthening. Roots soaked in water and crushed can be orally administrated to treat diphtheria [29,34]. *A. tortilis* also has been used for treating gastrointestinal disorders especially described for camelids. Their seeds, suckers, stipules and young spines have been found to be a remedy against sand colic that mostly to dromedaries. Moreover, its chewing gum has wound and burns healing properties, and when seeds or bark from *A. tortilis* are mixed with seeds from *Vigna unguiculata*, this mixture can be applied for treating skin issues (edema or allergic dermatitis) or as antiparasitic, respectively [34]. Pods from other species, like *A. arabica* or *A. catechu*, have also been reported for confectioning fodder for animals, particularly for sheep and goats [33,77]. In fact, *A. arabica* has been widely utilized in humans as a treatment for multiple affections and diseases, very similar to those already cited. The bark is considered a powerful astringent, and its extract has been used to allay irritation in acute gonorrhea and leucorrhea, cystitis, vaginitis and anal or uterus prolapsed. Decoctions or dry powder were used for treating hemorrhages, skin wounds, ulcers or leukoderma, diarrhea, dysentery, leprosy, diabetes, bronchitis, seminal weakness, as diuretic or anthelmintic agent. Noteworthy, its leaves have also been used for diarrheal disorders. Gargles were applied for cancerous and syphilitic affections, sore-throat, cough or toothache since it has been described as tonic, demulcent, aphrodisiac and anti-viral. The ground bark of *A. arabica* mixed with seeds of *Sesamum indicum* have been used as food and the juice of their bark mixed with milk is dropped into the eye for treating conjunctivitis. Pods, fruits, flowers, roots, leaves and gum present very similar applications; additional ones include the treatment of eczema and abscess with leaves, the use of fried gum for preparing sweetmeats or flowers as antipyretic. Moreover, the gum obtained from this species can be fried using ghee, a kind of clarified butter traditionally confectioned in India, for preparing sweetmeats and roasted seeds which served as food during acute scarcity periods [33]. Bark decoctions of another species, *Acacia catechu,* also has been reported to cure cold and cough, severe diarrhea or piles (applied with lemon slice), as tonic for women after delivery (with cardamom) while heartwood can be used as antipyretic, for cold during the pregnancy and to cure ulcers both in skin and mouth/tongue [77].

Several plants have been used in labor and delivery, such as the fern *Asplenium ceterach* (accepted name of *Ceterach officinarum*), used in cows and ewes after delivery as depurative. This effect is attributed to the astringent tannins present in its composition [68]. Another example is the plant *Capsella bursa-pastoris*, known as *shepherd’s purse*, which presents anti-hemorrhaging properties associated with the presence of tannins [78]. In this case, a decoction of the plant was given to pregnant animals to avoid hemorrhage [68].

Different pharmacopeias worldwide, including Russia, India and some European countries (France and Deutschland), have described the healing properties of several tannin-based rich plants like *Betula* species. Most texts point to bark as the main plant target to prepare decoction- or infusion-based extracts. Nevertheless, other parts like leaves, flowers, stems, roots or even sap or resin have also been exploited. *Betula pendula* is the species that has been further used and reported to have pharmacological properties. The principal applications of the extracts of *B. pendula* are aimed to treat or prevent urinary affections such as infections of the urinary tract or bladder, renal inflammation, renal stones or hindered dieresis. It also has been widely used for treating systematic diseases (rheumatism or arthritis), blood system disorders, respiratory tract ailments, or as wound healing, antipyretic or even anti-alopecic agent. Other minor utilizations included it as a remedy for spleen affections, hypercholesterolemia, headache or even as anti-helminthic [35]. Betulinic acid, a triterpenoid acid extracted from *B. pendula*, is well-known for its antiviral, tested against HIV, and anti-inflammatory activity, which may act in synergy with the tannins present [36].

*Castanea sativa* has been referenced as alimentary or medicinal with applications as laxative or stomach regulator when chestnuts are consumed or even as hemoptysis agents. *C. sativa* episperma was, however, described as astringent. Nevertheless, its most relevant value has been underlined as a source of nutrients even though it also has been cited as a possible antidote to lip and esophagus lacerations caused by *Colchicum autumnale* poison [79].

Edible nuts from *Juglans regia* had been recognized since ancient times, but also other tissues were exploited with diverse purposes, such as its leaves, which have been used to wrap cheese in order to provide aroma, but also antiparasitic properties [38,42]. Moreover, it has been traditionally applied as an anti-inflammatory for rheumatism and hemorrhoids, antipyretic, antifungal, antitussive and for skin affections. Specific applications of each plant part include bark decoction to gargle it for toothache, direct application of fresh leaves to reduce varicose veins, mild skin inflammations and its use as a local analgesic, and immature fruits to color hair. Moreover, a dosage of one teacup of *J. regia* twice or three times a day during one month has been described to be able to provide antidiabetic, hypercholesterolemic (HDL cholesterol), cardiotonic and vasodilator properties and reduce infecundity [37,38]. In fact, scientific reports have described tannins from walnut to possess anti-platelet, cardioprotective, antiatherogenic and anti-inflammatory properties [39,40,41].

The genus *Lotus* has been traditionally utilized as forage for different ruminants [80,81]. *Lotus* species, rich in CT, have been demonstrated to provide different benefits, such as favoring the weight gain, the growth of wool, improvement of the production and composition of milk and reducing the number of anthelmintic products in farming animals [81]. In cattle, the production of methane was reduced and enhanced ruminal fermentation when the animals were fed with *Lotus corniculatus*, which was attributed to the CT [82]. The administration of *L. corniculatus* has shown positive effects on milk production and gastrointestinal function of sheep [80]. In addition, *L. cornicatus*, *Lespedeza cuneata* and *Hedysarum coronarium* have shown anti-helminthic effects on ewes, reducing the presence of fecal eggs and worms and inhibit the development of larvae [83,84].

The genus *Phyllanthus* has a long clinical application in Asia. The fruit of *P. niruri*, *P. amarus*, *P. fraternus*, *P. debilis* and *P. maderaspatensis* and the leaves of *P. polyphyllus* has been applied for their tonic properties to liver diseases such as jaundice in India while for urinary affection were used *P. simplex*, *P. reticulatus*, and *P. acidus*. In India, these plants have also been utilized for wound healing, as antipyretic, anti-inflammatory or for treating diabetes. Similarly, this genus has been used as antipyretic and antitussive in China and for treating blood, bile disease, hypertension and anuria in Tibetan medicine. *P. urinaria* has been described to possess detoxifying properties and was also used for liver-based diseases (jaundice or hepatitis B), gastrointestinal (enteritis, diarrhea), or systematic affections (dropsy). As in India, other species like *P. reticulatus, P. niruri* and *P. simplex* were applied for treating urinary infection, among other inflammatory processes like rheumatism. In Thailand, *P. emblica* is used as the previously cited “Triphala” for chronic gastrointestinal diseases. However, other species are utilized for treating the same affections *P. amarus*, *P. urinaria* and *P. virgatus* are aimed at treating liver diseases, diabetes or gonorrhea. *P. acidus* was the remedy for slightly different affections like hypertension, constipation, fever or skin issues, whereas urinary infections or malaria are treated with *P. taxodiifolius*, *P. niruri*, and *P. reticulates*. However, in Africa, *P. muellerianus* is the most used species, and the one applied for malaria (and *P. reticulates*), tetanus, as antipyretic and wound healing. Instead, *P. polyanthus* is applied in Kenya for treating sexually transmitted diseases. Similar applications are found in South America, where leaves of *P. tenellus* have diuretic properties, *P. amarus* and *P. sellowianus* are used for treating diabetes, but also for jaundice and urinary infection, respectively [48]. For *P. niruri*, different bioactivities have been demonstrated, such as antioxidant, anti-inflammatory, anti-nociceptive, analgesic, hypoglycemic, lypolipidemic and hepatoprotective [49].

Different *P. abies* plant parts have been also applied in traditional culinary art. Young sprouts are used as food ingredients, while leaves (needles), flowers, pinecones and resin are utilized as food supplements. A Finnish novel food called “pettu” is prepared from the bark that is roasted and scratched of oozed substances or just boiled for 2 to 3 h. Then, the bark is dried, grounded and mixed with some cereals-based flour at equal parts since the consumption of pure bark can induce stomachache and constipation. This product can be used as bread-preparing flour; it can also be mixed with milk or animal fat, or blood or to prepare soup to where it provides a thickening effect [43]. This polyvalent flour was used in the 1860 s, during the famine, in Finland [85]. Other uses of different tissues of *P. abies* include animal treatments. Twigs serve for feeding calves, resin heals skin afflictions, and sores and ointments were used to treat or prevent respiratory affections like cough or pneumonia. This ointment of *P. abies*, when combined with other species like *Rumex obtusifolus* provides a remedy for mastitis [86]. Indeed, the veterinarian use of *P. abies* has been recognized by the European Medicines Agency through Veterinary Medicines and Inspections. The final preparation to administrate to animals is named *Piceae turiones recentes extractum* and can be obtained from boiling 10 to 15 cm long shoots, collected in spring, of fresh *P. abies.* This extract is then mixed with starch and an herbal powder. The final product that can be orally administrated (dosage: 0.6–6.4 mL solution, equivalent to 3.1 mg to 30.6 mg spruce-tips extract, per kg body weight) is aimed to treat diarrhea in cattle, horses, pigs, sheep and poultry [87].

Plants such as *Parietaria officinalis*, *Pistacia lentiscus*, and *Prunus spinosa*, *rupestris*, also present tannins in their chemical composition, have been traditionally used to treat different disorders of domestic animals. The main use described for *Parietaria officinalis* is the treatment of diarrhea in domestic animals. *Pistacia lentiscus* and *Prunus spinosa* present a high content of tannins, and they have been reported to exert beneficial effects on the protein metabolism of ruminants, improving the absorption of amino acids in the small intestine [47]. *P. lentiscus*, known as lentisk, has also shown antiparasitic activity against intestinal helminths and coccidia on sheep and goats, which have been attributed to the presence of tannins and other compounds [44,45]. Additional traditional uses of this plant include the treatment of scabies, diarrhea, constipation, dermal affections and infected wounds [46,88]. In the case of *P. spinosa* or plum tree, this plant has also been applied externally to treat wounds infected by worms [46] and also to control diarrhea [89]. Similarly, *P. officinalis* was also effective in controlling diarrhea, which is attributed to the presence of astringent compounds such as tannins [68]. In addition, this plant was used to elaborate tisanes with anti-inflammatory and antiseptic properties in combination with other traditional plants, such as *Pisum sativum*, *Beta vulgaris*, *Lavandula latifolia* or *Malva sylvestris* [61].

*Punica granatum* has been used as itself or in combination with other plants to treat very variable affections. When used as a unique herb, mainly bark and roots were used to treat intestinal worms, decoctions of pomegranate hulls were described as strong astringents and a remedy for treating dysentery, diarrhea, and stomatitis. Pomegranate hulls and/or root extracts were also administrated orally and intravaginally to minimize fertility and treat gynecological affections. Alternative uses include the application of pomegranate extracts for snakebite, diabetes, burns and leprosy, while the fresh fruit has been used as a refrigerant to ameliorate fever processes [90,91]. When used in combination, very assorted plants were used such as *Achillea millefolium*, *Artemisia* sp., *Emblica officinalis*, *Nepeta* sp., *Tanacetum* sp., *Taraxacum officinale*, *Terminalia chebula*, or *Zingiber officinale*, among many others. These mixtures of plants were mostly administrated to treat cold, cough and fever [92].

Among the genus *Quercus*, few examples of traditional medicinal or pharmacological uses can be found in the literature. A decoction of roots from *Q. cerris* and *Q. coccifera* can be applied as lotion twice a day for 2–3 weeks to treat skin burn, boil and wound. Fruits of *Q. coccifera* are edible and can represent a remedy for controlling diabetes. A teacup of a decoction of seeds from *Q. ithaburensis* administrated 2–3 times a day for one week can improve cold and flu processes [37]. A decoction of oak-apples from *Q. pubescens* together with other plants like mallow or chamomile was also used for healing wounds in newborn infants [42]. Tannins from *Quercus* had been described to have antioxidant and anti-diabetic activity [50,51,52].

Several species belonging to *Rubus* have been considered for their properties in different traditional medicines. Bud, fruits, leaves and roots of *R. canescens* can be eaten raw or drunk as an infusion (teacup twice a day for 2–3 weeks) or applied as a decoction for treating gastrointestinal issues (as carminative, dyspepsia and intestinal spasm) or diabetes. Similarly, fruits of *R. idaeus* can be eaten twice or three times a day for 2 to 5 days for mouth sores and as antiemetic. However, *R. sanctus* has wider applications, including treatment for atherosclerosis, stomachache, diabetes, eye diseases, nephralgia, kidney gravels, rheumatism, cold and flu, bronchitis, burn, boil and wound care, or as anti-hemorrhagic [37]. Leaves from *R. ulmifolius* can be topically administrated for hemostatic for cuts and for removing thorns; the extract of tender tips is useful for skin cuts and bruises, and jam obtained from fruits relief cough and sore throat. Moreover, *R. ulmifolius* sprouts can be mixed with walnut kernels, *Verbena officinalis* leaves, *Sambucus nigra* bark, cyclamen tubers and bramble buds to prepare a cream with beeswax and oil basis that can be administrated for inflammation processes [42].

*Rhus* genus has been traditionally applied and named as sumac. Its main uses included it as a food condiment, but also for the treatment of gastrointestinal diseases such as diarrhea, intestinal ulcers, rectal prolapse and hemorrhoids, oral diseases, dysentery, or stroke. Sumac has been described to possess anti-inflammatory, immunomodulatory, antimicrobial, antiviral, antioxidant, antifungal and antiapoptotic effects [53,54].

Several species from the genus *Sapium* have been described to be used as part of traditional medicine in different cultures. Most of the *Sapium* species were used to treat skin-related diseases such as eczema, dermatitis, wounds or snake bites. However, additional uses were pointed to this genus as a remedy for overstraining, lumbago, constipation and hernia. For instance, *Sapium baccatum* has been used for treating eczema, roots bark and leaf from *Sapium japonicum* were applied for treating overstrain, lumbago and knee pain and similarly, the resin from *Sapium glandulosum* was used for hernias. Other uses included the treatment of digestive and urinary ailments (*Sapium sebiferum* root bark and seed), skin affections and antiparasitic (bark juice of *Sapium insigne*). Different plant tissues of *Sapium ellipticum* were applied with different purposes such as for respiratory complications (root decoction and dried stems), abdominal swelling, eye diseases and mumps (leaves), malaria (root decoction), anemia, fever, guinea worms, elephantiasis and rheumatic problems (stem bark decoction) [93].

*Smilax aspera* or sarsaparrille, another plant that has been reported to contain tannins in its chemical composition, has been administered orally as a diuretic and urinary antiseptic in cows and has been used in the alimentation of rabbits due to its beneficial effects on the health of the animals [61]. In addition, decoctions of this plant were applied to eliminate purulent vesicles [47].

To our knowledge, the traditional uses of the *Schinopsis* genus as medicinal and pharmacological remedies are few. Leaves, bark, resin and fruits from *S. brasiliensis* were used in the popular medicine as a general anti-inflammatory, for treating cold, cough, fever, diarrhea, dysentery, fractures and as antimicrobial [55]. *S. lorentzii* has been reported to be traditionally used for treating stomachache, headache or cough when prepared as infusion or decoction using leaves and tender branches. Nevertheless, leaves have been described to be useful as a cicatrizing agent and to relieve bruises while the bark may have anti-asthmatic properties [56,57]. The cortex of *S. balansae* also has cicatrizing properties accompanied by anti-inflammatory and antiseptic capacity. The wood of *S. balansae* has been referred to as astringent, and fresh sap may remove moles [57]. The main properties associated with *Schinopsis* are antioxidant, antimicrobial, anthelmintic [58,59,60].

Similarly, in the case of the genus *Terminalia*, many species had been contemplated as medicinal plants such as *Terminalia bellirica*, *T. chebula*, *T. arjuna*, *T. catappa*, etc. Among them, *T. bellirica* has been studied for being considered edible and for its multiple properties to treat edema, diarrhea, leprosy, bile congestion, indigestion, headache, fever, cough, dysentery or skin diseases [94]. Different plant structures have been suggested to have diverse applications. For instance, fruits can be utilized for respiratory tract affections like cough (decoction), hoarseness, asthma or bronchitis; for digestive issues (indigestion, diarrhea, edema or hemorrhoids that can be treated with pulp fruit), menstrual disorders, hepatitis, as purgative or even as a hair tonic. Fruit kernel has been described as narcotic, and its oil was purgative like bark gum. Seed oil has anti-rheumatic activity while leaves improve health status by improving immunity, acting as anti-aging, enhance appetite, relieve hemorrhoids and can reduce cholesterol and blood pressure [70]. Extracts obtained from the bark of *T. arjuna* are aimed as cardioprotective and antihyperlipidemic, but also as a remedy for muscle sores, contusions, fractures, ulcers, treatment of bile infection, dysentery or as poison antidote [94,95]. For *T. chebulla*, the fruit has also been widely used for digestive alterations to improve appetite, as an astringent, antiemetic, stomach tonic, mild laxative, for hemorrhoids or as antispasmodic [96]. *T. chebulla* can also be applied for infertility, asthma, sore throat, dental caries, urticaria, dysentery, bleeding, ulcers, gout and bladder disease [94]. A paste obtained by mixing grounded *T. chebulla* with water has anti-inflammatory, analgesic and wound healing capacities while its decoction helps to treat oral ulcers or sore throat [96]. A combination of dried fruits obtained from these two species, *T. chebula* and *T. bellirica*, and *Phyllanthus emblica*, is known as “Triphala,” being long-used as a restorative and revitalizing natural formulation that boosts the immune system against infectious diseases [70].

The nutritional value of *Urtica dioica* has been recognized from ancient times for both humans and animals. It was administrated as a revitalizing agent for humans [63]. In animals, it is also restorative and promotes weight gain and growth of chicks, Turkey cocks and pigs, and it was also described to increase the production of galactagogue (a substance that promotes lactation) in ruminants [63,66,68]. Currently, *U. dioica* is consumed as part of curry, soup, vegetable complement or as the main ingredient of an omelet. Within its chemical composition, tannins have been quantified in 0.93 mg/100 g, 38% of proteins, 9% of crude fiber and 0.2% for both calcium and iron. The fresh plant has been applied both directly and as an infusion for treating arthritis, lumbago, rheumatism, muscular or limb paralysis. The direct use of *U. dioica* has been described as a rubefacient; thus, it was utilized for the stimulation of blood circulation, which helped the warmth of joints and extremities. This plant was also stated to relieve symptoms of allergic rhinitis and to provide vitality to people [63]. Indeed, scientific works have described antioxidant, anti-inflammatory, antimicrobial, analgesic, antidiabetic, and antimutagenic activities for *U. dioica* extracts [64,65]. Another plant used in animal feeding is *U. dioica* (traditionally named nettle), which has been given to chicks, Turkey cocks and pigs as restorative to promote weight gain and the growth of the animals [66,68]. It has also been described to increase the production of galactagogue (a substance that promotes lactation) in ruminants [67]. Different compounds have been identified in *U. dioica*, including tannins [46].

The traditional veterinarian applications of the species *Umbilicus rupestris,* also known for its common name navelwort, are well documented. This plant, which has been described to possess tannins, has been used externally to treat wounds of animals [61,62] and orally to control diarrhea, fever, intoxications [47] and has been used as an antiparasitic in hens [61].

*Vitis vinifera* has been widely and repeatedly used as food and beverage ingredients, and its consumption has been associated with different beneficial health effects. It has been used for gastrointestinal diseases; sprouts can be eaten as thirst-quenching, but also for treating headaches (as vinegar), colds (mixed with honey, cinnamon and cloves), and wine baths were used for children to strengthen them or to treat inflammations [42]. Most of their traditionally and currently exploited bioactivities are directly related to their high content in molecules with antioxidant properties such as proanthocyanidins and anthocyanins [97]. Among their recognized benefits, extracts obtained from seed grapes have been described to possess anti-obesity and antidiabetic capacity by downregulating the lipid metabolism. The administration of grape seed extracts rich in procyanidins revealed a high increase in the expression of several genes involved in β-oxidation, involved in lipid catabolism, and which ultimately suggests their potential to prevent fat accumulation [98]. These extracts demonstrated to increase energy expenditure, and thermogenesis was also found to diminish the expression of TNF-α, a proinflammatory factor augmented in chronic diseases, such as obesity [99]. Procyanidins present in *V. vinifera,* besides inhibiting fat gain, have shown the potential to alter the small intestinal gut microbiota. This has been suggested to be related to their capability to improve glucose tolerance and insulin sensitivity, and hence described as antidiabetic [100]. Other tannin classes present in several species that possess antidiabetic or hypoglycemic properties are valoneic acid dilactone, TGG and PGG from *C. sativa*, both HTs (TGG, PGG) and CTs (catechin derivatives) from *S. lorentzii* or chebulanin, chebulagic acid and chebulinic acid from *T. chebula* [59,101,102].

Another source of tannins recently demonstrated is brown macroalgae with a high content in phlorotannins. Macroalgae have been used for nutritional purposes since ancient times, especially in the Far East Asiatic cultures, such as *Sargassum* and *Ecklonia*. *Sargassum,* among other algae species, has been historically used as edibles and folk medicine. Seaweed consumption was supported for its nutritional value but also because its regular ingestion was related to an effective reduction of depressive symptoms during pregnancy and with a diminution of suicide rate in Japan, while in Korea was associated with minor diabetes mellitus incidence. Additionally, these algae have been used as hydrocolloids, emulsifiers and gelling agents in various food product preparations [71,74]. Different species belonging to the genus *Sargassum* have been used in traditional Chinese medicine. *S. pallidum*, *S. confusum*, *S. fusiforme*, *S. fulvellum*, *S. siliquastrum*, *S. thunbergii*, *S. muticum* or *S. hornerii* have served as a treatment for goiter, inflammation-based diseases like scrofula, arteriosclerosis, hepatosplenomegaly or testes swelling, dropsy, edema due to retention of phlegm and morbid fluids, dysuria, respiratory affections like sore throat, cough, acute esophagitis or chronic bronchitis, angina pectoris and high blood pressure, skin diseases like furuncle, and even neurosis [71]. The potential mechanism through which *Sargassum* may exert its activity includes its capacity as antioxidant, antibacterial, antiproliferative, and anti-inflammatory [72,73]. *Ecklonia cava* is a highly valued edible brown seaweed in Japan, China and South Korea, where it is consumed daily. It is foremost intake as part of salads, miso soup, or powdered as a condiment in rice cakes, candies or kimchi [103]. It is recorded in Chinese Pharmacopoeia as part of preparations with other seaweeds like *Sargassum* sp. as “Laminaria Thallus”, and it is attributed to attenuate goiter, diuretic and treatment for mammary hyperplasia [71]. Additionally, it is allegedly held as “health-promoting” in Korea [104]. *E*. *cava* ethanolic extract, highly rich in PT (> 90%), has been approved for use as a food ingredient by both Food & Drug Administration (FDA) and European Food Safety Authority (EFSA) [75,76]. This approval is justified on the evidence regarding its antioxidant, antiviral or anti-inflammatory, as well as antidiabetic and anti-obesity properties [105,106]. In fact, an in vivo experiment determined significantly lower carbohydrate absorbance and metabolization in rats when administered said *E*. *cava* extract [107].

**Figure 2:**
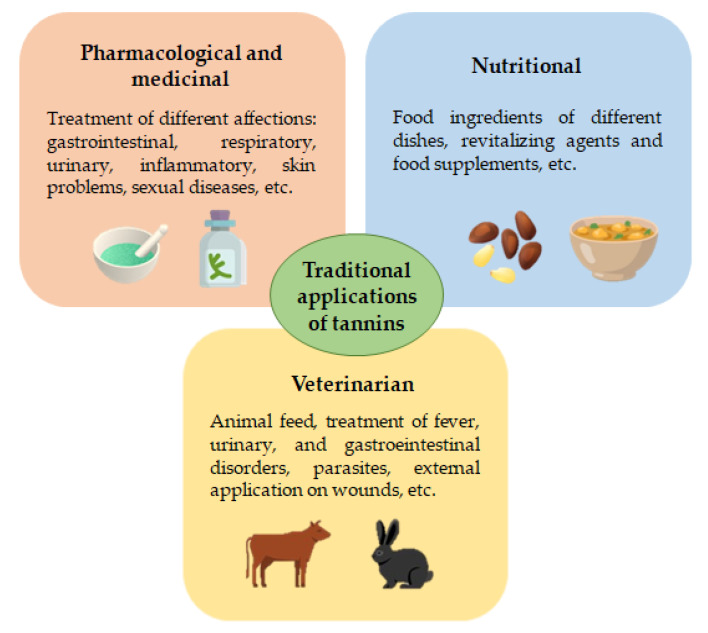
Brief description of the traditional uses of tannins with pharmacological, medicinal, nutritional, and veterinarian applications.

As a final mention to this section, in Figure 2, an illustrative description of the traditional uses of tannins with pharmacological, medicinal, nutritional, veterinarian and botanical applications is briefly depicted.

## 3. Chemical and Quantitative Composition of Rich Tannins Plants Traditionally Used

In traditional medicine, it is of great pharmacological interest to study the composition of plant extracts due to the direct relationship among chemical structures of compounds and their beneficial effect [108]. Plant polyphenols are suggested to exert their bioactivities in synergy and to be present in tannin extracts; therefore, some of these relevant compounds are also reported to offer a complete view of the beneficial potential of plant extracts [109]. Furthermore, some non-tannin molecules like several flavonoids, phenolic acids or phenolic glucosides have also been suggested or evidenced to contribute to polymerization or conformation of several tannins.

**Figure 3:**
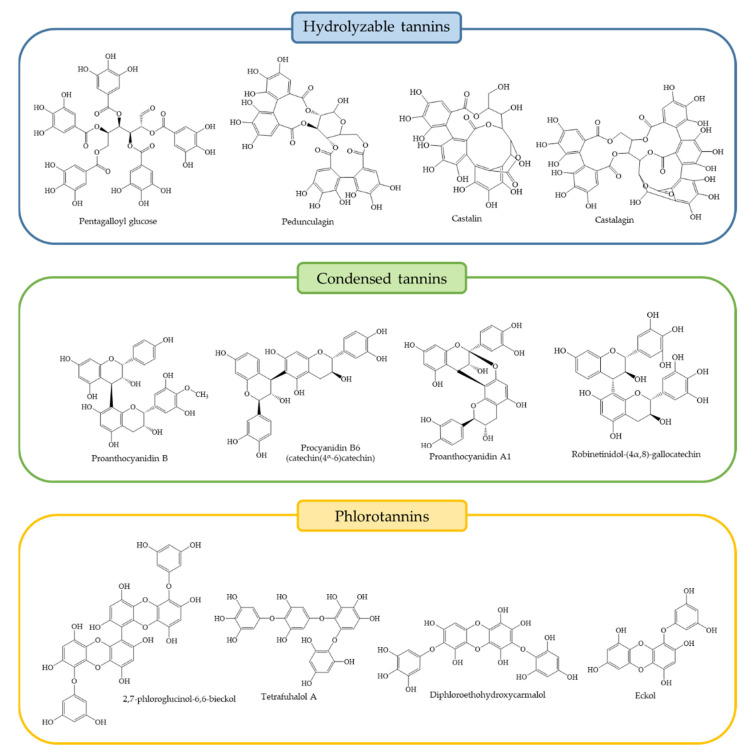
Chemical structure of main tannins present in the selected species.

Therefore, in the present section, we describe the chemical composition of plant species used in traditional applications (Table 2), the molecular structures of representative tannins of each structural group (Figure 3) and their quantitative presence in different species (Table 3).

There are hundreds of species of the genus *Acacia* globally distributed. Among them, *A. nilotica* has been widely documented for its traditional uses. The highest tannins levels are located in their fruits (22%), while leaves and bark account for half of this quantity [113]. Among the tannins identified in *A. nilotica* some of the most representatives are methyl gallate and polygalloyl units like ethyl gallate1-galloyl-β-D-glucose, 1,6-di-galloyl-β-D-glucose, gallocatechin-5-gallate, epigallocatechin-7-gallate and -5,7-digallate, diGA, dicatechin, and the phenolic acids GA and EA [30]. The tannin fraction of *A. mearnsii* is also of interest for pharmacists, and in fact, it is commercially available as a food supplement after hot water extraction. The composition is based on flavan-3-ols units, mainly the monomers fisetinidol, robinetinidol, quercetin, myricetin, catechin and gallocatechin, among others. They are linked by C–C bonds, so that the resulting polymeric flavonoids are bioflavonoids like fisetinidol-(4α-8)-catechin and robinetinidol-(4α-8)-catechin androbinetinidol-(4α-8)-gallocatechin and triflavanoids such as robinetinidol-(4α-8″)-robinetinidol(4′α-6″)-gallocatechin and robinetinidol-(4α-8″)-robinetinidol (4′α-6″)-catechin [111]. Similarly, *A. arabica* has been described to possess a 12–20% of tannins in the bark, accounting for these some tannin units like GA, (+)-catechin, (+)-catechin-5-gallate, (-) epicatechin, (-) epigallocatechin-7-gallate and -5,7-digallate, (+)-dicatechin or pyrocatechol. Fruit also contains 32% of tannins identified as oligomers or as structural units (digallic and GA with its methyl and ethyl esters, protocatechuic and EA, leucocyanidin, 3,4,5,7-tetrahydroxy flavan-3-ol, 3,4,7-trihydroxy flavan 3,4-diol and 3,4,5,7-tetrahydroxy flavan-3-ol and (-) epicatechol) [33]. *A. catechu* leaves possess high amounts of esterified monomers, such as epicatechin- and epigallocatechin-3-gallate, and some flavonols like quercetin and kaempferol. Aqueous extracts were characterized for containing rhamnetin, 4-hydroxyphenylethanol or profisetidin [110].

The genus *Betula* also contains a hundred species worldwide distributed; however, pharmacologists mainly focus on *B. pendula.* In general terms, *B. pendula* possess a wide range of phenolics, mainly glycosylated flavonoids and salicylates and high concentrations of CT (oligomeric and polymeric flavan-3-ols) [114]. In particular, *B. pendula* from southeastern Finland possess tannins and flavonoid-aglycones (apigenin, luteolin and chrysoeriol derivatives) [115]. Other work focused on *B. pubescens* collected from the same country revealed the content of CT and quercetin, apigenin, naringenin derivatives, kaempferol and myricetin derivatives [131].

Bark, resin, fruits and/or leaves of two common trees have been widely applied as traditional remedies and are well known to contain tannins. This is the case of the genus *Castanea,* being the most common representative *C. sativa.* Organic and aqueous extracts obtained from burs of *C. sativa* were evaluated with HPLC-UV-HRMS (high-performance liquid chromatography coupled to ultraviolet high-resolution mass spectrometry). Chromatograms from chestnut revealed that the most relevant tannins present in *C. sativa* are EA (5–79 mg/g) and chestanin (1–13 mg/g). However, the presence of many other several HT has been repeatedly demonstrated, such as trimethyl-ellagic acid hexoside or deoxyhexoside, chestanin, chesnatin, isochesnatin, cretanin, castalagin/vescalagin, methylvescalagin, pedunculagin, stachyurin or casuarinin, tellimagrandin I, chebulagic acid, castavaloninic or vescavaloninic acid, cocciferin d2 and castacrenin A-C isomers [101,116,117,118]. Other molecules described in chestnuts include ETs such as castalin, acutissimin A and B, grandinin or to its isomer roburin E, valoneic acid dilactone, tannin T1 and T2, MGG, TGG, TeGG, PGG, and HHDP-glucose derivatives (pedunculagin, casuarinin or tellimagrandin I) as well as the phenolic acid and GA [101,116,118]. Other minor compounds, present at trace levels, are 5-galloylhamamelose, (3,5-dimethoxy-4-hydroxyphenol)-1-β-D-(6′-o-galloyl)-glucoside isomer, m-digallic acid and kurigalin isomer [117].

Walnuts obtained from *J. regia* were analyzed in terms of polyphenol content. The main phenolic compounds were ET, even though they are more abundant in seeds, together with GT. Many of these HT found in *J. regia* were identified through the presence of the HHDP group. HT present in *J. regia* were tentatively identified using an LTQ-Orbitrap based on MS/MS results. The major ones semiquantitatively determined were EA and HHDP-glucose. Many derivatives of the latter one were suggested to be present such as pedunculagin or casuariin (bis-HHDP-glucose), valoneoyl, sanguisorboyl, tergalloyl or macaranoyl (HHDP-glucose+trigalloyl group), tellimagrandin I (digalloyl-HHDP-glucose), precoxin A and its isomers such as flosin A, platycariin or platycaryanin B (trigalloyl-HHDP-glucose), glansrin C, alnusnin B, asuarinin/casuarictin isomers (galloyl-bis-HHDP-glucose), strictinin/isostrictinin isomers (galloyl-HHDP-glucose), stenophyllanins A-C, malabathrin A, eucalbanin A or its isomer cornusiin B, heterophylliin E, pterocarinin, breginin A (dimer of casuarinin and pendunculagin) and alienanin B (dimer of casuarinin and stachyurin), and flavogallonic acid dilactone [39].

More than 500 compounds have been isolated from *Phyllanthus*, although tannins together with lignins are considered the main bioactive compounds of this genus [48]. The species *P. niruri* contains ETs (HT) and flavonoids (CT). It is worth mentioning that the final hydrolysis of ETs gives the phenolic acids EA and GA, and many of them display activity against some viruses [132].

The characterization of the chemical profile of aqueous extracts of four *Phyllanthus* species (*P. amarus*, *P. stipulatus*, *P. niruri* and *P. tenellus*) have revealed C-glycosylated flavones, O-glycosylated flavonols and the ETs geraniin A and B, phyllanthussin C, pelargoniin A, chebulagic acid A and geraniinic acid A. Moreover, the flavonol quercetin-3-O-β-D-glucuronopyranoside was purified from the aqueous extract of *P. stipulates* [133]. It was also reported the isolation of corilagiri, a tannin with antihyperalgesic activity, from the specie *P. niruri* [134]. The HT have been shown as the main therapeutically active molecules of *P. amarus* [132], including different kinds of ETs: geraniin, amariin, furosin, geraniinic acid B, amariinic acid, amarulone, repandusinic acid A, corilagin, isocorilagin, elaeocarpusin, phyllanthusiin A, B, C, D and melatonin [135]. *Phyllanthus phillyreifolius* var. *commersonii* from Mauritius has also been documented as a potent source of bioactive phytochemicals. Its aqueous and methanolic extracts are abundant in ETs (including phyllanthusiin B and granatin B), while phenolic acids, GA and flavonoids, quercetin and derivatives, were also present [136].

Bark, resin, fruits and/or leaves of two common threes that have been widely applied as traditional remedies and are well known to contain tannins are the genus *Picea*. One of the most utilized species for treating human and animal affections is *P. abies*. Leaves of *P. abies* have been analyzed and have shown to contain mono-terpenes (bornyl acetate, α-pinene, camphene, and limonene), di-terpenes (manool and dehydroabietate), sesqui- and triterpenes; phenolic compounds (CT; flavonoids like kaempferol, quercetin, myricetin; stilbenes and lignans) and alkaloids. The group of phenolic compounds represents 23% without counting the 25% the group of the CT, triterpene saponins/glycosides are a 3%, essential oils account for 2% and flavone glycosides [87]. The resin of *P. abies* is a very useful product that may account for up to 19 g dry plant equivalent/100 g finished product [86].

The traditional medicinal plant *P. lentiscus*, typically found in the Mediterranean countries, has revealed at least 46 compounds, including flavonoids, hydroxycinnamic acid derivatives, phenolic acid derivatives and other polar compounds. Among them, the most prevalent flavonoids are catechin, myricetin galactoside, myricetin galloy, rhamnopyranoside, myricetin-xyloside, myricetin-rhamnoside isomers and quercetin glycoside and the phenolic acid derivatives comprised galloyl quinic acid, digalloyl quinic acid, digalloyl quinic acid, dallic acid methyl ester and pistafolin A [137]. In addition, active components like polyphenols, flavonoids, A-type proanthocyanidins, anthocyanins, coumarins and phenolic acids were identified in *P. spinosa* [138].

An ethanolic extract obtained from the wood of *Q. robur* was analyzed and observed to possess HHDP−glucose, castalin, vescalin, vescalagin, castalagin, grandinin or to its isomer roburin E, polygalloylglucose isomers (TGG, TeGG, PGG isomers), and triterpenoid glycosides, among others like triterpenoid or polygalloylquinic acid (tri-, tetra-, penta-, esa-galloylquinic acid) derivatives, as well as the phenolic acids, EA and GA [50]. Bark from *Q. petraea* has been characterized to contain ETs such as 2,3-(S)-hexahydroxydiphenoyl-glucose, pedunculagin, vescalagin, and castalagin; the flavanoellagitannins acutissimin A, acutissimin B, eugenigrandin A, guajavin B, and stenophyllanin C; and the procyanidin ET [139].

*Rhus coriaria* mainly contains HT that, in general terms, have a common chemical base, the GA unit. Some works described its content as GA, quercetin-glucoside or cyanidin-3-glucoside equivalents and described its cyaniding-derivatives content as follows: cyanidin-3-(2″galloyl)-galactoside, 7-methyl-delphinidin-3-(2″galloyl)-galactoside, methyl delphinidin aglycone, 7-methyl-cyanidin-3-(2″galloyl)-galactoside [54]. Among the HT present in *R. coriaria,* it is worth mentioning galloylhexose, benzoic acid, 3,4, 5-trihydroxy-, 2-oxo-1,3-propanediyl ester, galloylnorbergenin isomers, digalloyl-hexoside, galloylshikimic acid, methyl digallate isomers, galloylquinic acid, quinic acid, isomers of trigalloyllevoglucosan, myricetin galloylhexoside, triGA, galloyl arbutin, arbutin, digalloyl-hexoyl-EA, pentagalloylhexoside, hexagalloyl-hexoside and dihydroxybenzoic acetate-digallate and galloyl-valoneic acid bilactone [123].

Extracts obtained from the seeds of *Rubus occidentalis* were demonstrated to contain EA derivatives and ETs such as sanguiin H-10 isomer, lambertianin C without ellagic moiety, sanguiin H-10 isomer, lambertianin C and sanguiin H-6, proanthocyanidin trimers, galloyl-HHDP glucose or galloyl-bis-HHDP glucose isomer [140]. The number of anthocyanins quantified in fruit extracts obtained from *R. idaeus* accounted for 5.7 mg/g (73% of total polyphenols), being the major ones cyanidin-3-sophoroside cyanidin-3-glucoside, cyanidin-3-glucosyl-rutinoside and cyanidin-3-rutinoside [141]. Similarly, the most abundant anthocyanin identified in the juice of *R. fruticosus* was cyanidin-3-glucoside, and as minor ones malvidin-3-galactoside, cyanidin-3-galactoside and delphinidin-3-galactoside. Other compounds present in the juice were the phenolic acids GA and vanillic acid or flavanones such as naringenin, but in much lesser amounts [124].

From *Sapium baccatum,* apart from the phenolic acid, GA, and the flavonoid, quercetin 3-α-L-arabinopyranoside, the following tannins have been identified: methyl gallate, corilagin, tercatain, chebulagic acid and chebulinic acid [125].

Among other relevant bioactive compounds such as fucoxanthin, sulfated polysaccharides (i.e., fucoidan), tocopherols or vitamins, the main PT described in *Sargassum fusiforme* or *S. horneri* are fuhalols, fucols, ethols, fucophloroetols and several carmalol derivatives [73]. Many studies have attempted to elucidate the specific structure of some of these phlorotannins by, i.e., HPLC/MS methods, but only main groups of PT are reported. However, research on other *Sargassum* species such as *S. muticum* or *S. spinuligerum* show colliding results regarding the main PT, which are fuhalols such as hydroxytrifuhalol B, hydroxypentafuhalol A, hydroxyheptafuhalol B and hydroxynonafuhalol A [126]. Conversely, structurally different PT like eckol derivatives have only been reported in algae from *Ecklonia* sp. and *Eisenia bicyclis* such as dieckol, 2,7-phloroglucinol-6,6-bieckol, phlorofucofuroeckol-A, pyrogallol-phloroglucinol-6,6-bieckol have been recently described in *Ecklonia cava* by HPLC [119].

Red quebracho species (*S. lorentzii* and *S. balansae*) are, as mentioned, one of the most common and abundant sources of CT (14–26% of heartwood), and commercial extracts are even employed as quantification standards (about 300 mg/g) [1,6]. Extracts are usually obtained from bark or heartwood and have been used since the 19th-Century for leather tanning, but also exhibit other industrial and bioactive properties. Quebracho extracts are generally composed of 95% CT and 5% polysaccharides. Of these CT, some authors have determined that they are almost entirely profisenitidin polymers, formed by fisenitidol extenders bonded through a catechin subunit by C4-C8 linkage [58]. In fact, recent work has described the presence of the following CT: dimer constituted by catechin-fisetinidol isomer, dimers of catechin-3-gallate and fisetinidol, trimers of catechin-3-gallate and two fisetinidol, trimers of one catechin and two fisetinidol units, tetramers of one catechin and three fisetinidol units, a tetramer of one catechin-3-gallate and three fisetinidol. Moreover, it also revealed some of the HT present in *S. lorentzii*: TGG and PGG isomer, as well as esters of quinic acid with different units of GA [59].

The genus *Smilax*, typically found in tropical areas of Asia and the United States, possesses more than 350 species. Moreover, finally, the NMR characterization of root extracts from *Smilax aspera* with antifungal activity have shown steroidal saponins, resveratrol (phenolic compound), curillin G, asparagoside E, asparoside A and asparoside B [142].

Other plants used in traditional medicine are those from the genus *Terminalia,* which comprises 200 species. These plants have been revealed the occurrence of several classes of tannins and pseudotannins. For instance, the species *T. chebula*, *T. bellerica* and *T. horrida* are rich sources of GA and other simple gallate esters like methyl gallate, 1,6-di-galloyl-β-ᴅ-glucose, 3,4,6-tri-galloyl-β-ᴅ-glucose, 1,3,4,6-tetra-galloyl-β-ᴅ-glucose and 1,2,3,4,6-penta-galloyl-β-ᴅ-glucose [95,128]. It was also reported the identification of chebulic acid and ET (chebulic and non-chebulic) in fruit and leaves from several *Terminalia* spp., being chebulanin, methyl neo-chebulanin, corilagin, punicalagin and terflavin the most frequent molecules. In addition, it is widely reported the isolation of EA and their derivatives flavogallonic acid, gallagic acid and methyl flavogallonate and the identification of several molecules from the EA glycosides group [95].

In the same way, antioxidants omega-3 polyunsaturated fatty acids, tocopherols, polyphenols, flavonoids, phenolic acids and a phenylpropanoid glucoside were characterized from hydroethanolic extracts of *U. rupestris,* which also showed antibacterial activities [143]. *U. dioica* is the unique species of the *Urtica* genus provided by the pharmaceutical industry. The powder from medicinal herbs includes phenolic acids, flavonoids, tannins, curcuminoids, coumarins and lignans, among others [63].

Mainly seeds and skin of *V. vinifera* have been analyzed to test the presence of tannins since they are the tissues with major abundances. While tannins from grape seeds are smaller in size due to a low polymerization degree, skin tannins are conversely heavier because of the higher polymerization degree. Seed tannins are mainly procyanidins polymers. Grape skin mostly contains epicatechins, which can be present as epigallocatechin, catechin dimers (procyanidins B) or catechin-gallocatechin dimers, but it also possesses fisetinidin dimers [129,130].

**Table 3:** Important species as sources of tannins. Quantitative data of hydrolyzable and condensed tannins (expressed in mg/g of dry weight (dw), except when other units were indicated) determined using different detection methods in specific tissues of the indicated species.

Species	Tissue	Type	Method	Concentration (mg/g dw)	Ref.
*Acacia* sp.	Leaves	HT, CT	Folin–Ciocâlteu	84–256	[144]
	Bark	CT	HPLC-UV-MS	108	[145]
*Betula* sp.	Leaves	CT	Abs. 550 nm	73–81	[115]
*Castanea sativa*	Bark, heartwood, peel	HT	HPLC-DAD-MS	47.5–167.3 (bark), 62.8 (heartwood), 4.9 (peel)	[22,146,147]
*Ecklonia cava*	Whole alga	PT	HPLC	6.07	[148]
*Hedysarum* sp.	Whole plant	CT	-	68	[149]
*Juglans regia*	Seeds	CT, ET	-	35–87(CT), 36–59 (ET)	[5]
*Lespedeza procumbens*	Leaves	CT	Abs. 550 nm	60–130	[150]
*Lotus* sp.	Flowers, leaves, stems and roots	CT	Abs. 550 nm	25–54	[151]
*Parietaria* sp.	Whole plant	CT	Abs. 550 nm	10 mg DE/g dw	[47]
*Pistacia* sp.	Leaves	CT	Folin–Ciocâlteu	21.7–25.1	[152]
	Hulls	HT	HPLC-DAD-MS	20.4–33.1	[153]
*Prunus* sp.	Fruits and leaves	CT	Abs. 550 nm	2.2–37.6 (fruit), 74 (leaves)	[154,155]
*Punica granatum*	Whole fruit	HT	Abs. 550 nm	62.71–139.63 mg TAE/g dw	[156]
*Quercus* sp.	Whole fruit	HT	Abs. 270–325 nm	8.18–47.26	[157,158]
*Rhus* sp.	Leaves	GT	Folin–Ciocâlteu	13–550 mg GAE/g dw	[159]
	Plant	HT	LC–MS/MS	230.7 mg/kg	[160]
*Schinopsis* sp.	Barks	CT	Folin–Ciocâlteu	453 mg TAE/g dw	[161]
	Heartwood	CT	HPLC	164	[145]
*Smilax* sp.	Leaves	CT	Abs. 550 nm	11.36 mg DE/g dw	[47]
*Umbilicus* sp.	Whole plant	CT	Abs. 550 nm	5.45 mg DE/g dw	[47]
*Urtica* sp.	Whole plant	CT	Abs. 550 nm	8 mg DE/g dw	[47]
*Vitris* sp.	Skins and seeds	CT	Abs. 500 nm	6–165 mg CE/g dw (skins), 3–241 mg CE/g dw (seed)	[162]

TAE: tannic acid equivalents, GAE: gallic acid equivalents, DE: delphinidin equivalents, CE: catechin equivalents. HPLC: high-performance liquid chromatography, DAD: diode array detector, LC–MS/MS: liquid chromatography–tandem mass-spectrometry, UV: ultraviolet.

Finally, few different legume species, including *L. corniculatus*, *L. pedunculatus*, *Lespedeza* sp., *Leucaena* sp., *D. ovalifolium*, *Gliricidia sepium*, *Manihot esculenta*, *Arachis pintoi* or *Medicago sativa* L., have been repeatedly described to possess high amounts of CT. However, the relative information of the chemical profile of these molecules is not easily accessible [7,163,164,165,166]. Nevertheless, we have compiled the chemical profile data relative to the genus *Lotus* to provide an example of this family of legumes with traditional applications for veterinarian aims mainly. Two *L. corniculatus* varieties (Fergus and Viking) were deeply analyzed by MALDI-TOF MS. The MS spectra revealed the presence of tetramers to hexamers (consisting of procyanidin or catechin/epicatechin units) but also included oligomers up to the decamer range. The most abundant tannins in *L. corniculatus* are heteropolymers, containing both catechin/epicatechin (procyanidin, PC) and gallocatechin/epigallocatechin units (prodelphinidin, PD) or homopolymers (PDs were not detected as homopolymer). Even though most of the heteropolymers were constituted by one or two PD units, few molecules displayed a maximum substitution degree of three or four units [120]. Another work used HPLC-ESI-MS for examining the CT content in *L. corniculatus* and compared it against *L. pedunculatus*. The analysis was performed after a strong acid-catalyzed cleavage in the presence of PG or benzyl mercaptan. The average MW of CT for *L. corniculatus* is lower (1900) than for *L. pedunculatus* (2200), besides CT concentration is also double in the latter species. Whereas *L. corniculatus* has a high abundance of PC subunits (ratio PC:PD close to 8:2) with a strong presence of epicatechin units, *L. pedunculatus* mostly possesses PD subunits (ratio PC:PD of 2:8), which are mainly located in extender units. Terminal units differ from extender ones since they have a lower content of epigallocatechin and epicatechin [81].

Therefore, plants and extracts reported to be used as remedies for human and animal ailments or affections have been found to possess high concentrations in tannins, which given their reported bioactivities, can be suggested to be responsible for the alleged benefits of these species.

## 4. Bioavailability and Bioaccessibility of Tannins

**Figure 4:**
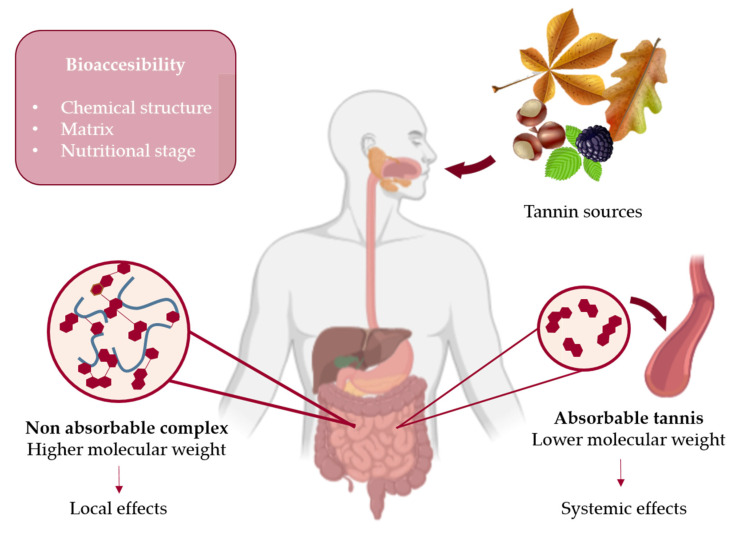
Bioaccessibility and bioavailability of tannins.

Traditionally, the most common application of rich-tannins plants has been through oral and topical administration, while as mentioned, several tannin-rich sources are commonly used as a foodstuff (i.e., *Sargassum* sp., *E. cava*, *C. sativa*, *P. granatum*, *V. vinifera*) (Table 1). The oral intake of tannins has two potential mechanisms of action; they may exert their biological effects as non-absorbable complexes or as absorbable simpler units. Non-absorbable complexes are created due to their binding properties that prompt their union with other present molecules in the organism, like proteins. These resultant complexes may produce local effects in the gastrointestinal tract (antioxidant, radical scavenging, antimicrobial, antiviral, antimutagenic and antinutrient). Absorbable tannins are simpler metabolites, characterized to have low-molecular-weight; thus, they are mostly dimers and trimers. These absorbable tannins can be present in the preparation administrated or can be the metabolic product of more complex structures that get fermented along the digestive tract. Either the origin of these absorbable tannins they are capable of reaching blood circulation and get transported into other organs, being then able to induce systemic effects [5,20]. Yet, despite evidence regarding tannin bioaccessibility on humans is scarce, some experiments have tested their absorbability in vitro and animal models. The bioaccessibility and bioavailability of tannins are both directly related to their chemical structure, the matrix into which they are embedded, but also with the digestibility capacity of the organism that intakes them. In general terms, highly polymerized tannins, with high molecular weight, are more poorly absorbed in the small intestine and rely on fragmentation and gut microbiota [167]. The conjugation of tannins with other molecules present in the matrix can enhance their transport as biotransformed polyphenols. For example, anthocyanins have been demonstrated to possess higher bioaccessibility when embedded into lipidic matrixes while protein ones prevent their degradation by small intestine conditions, which prompt their further metabolism at the colon, after which they can reach blood circulation [168] (Figure 4). This could be explained by the preferential absorption of lipophilic molecules by the gut, a feature that has able to enhance the bioaccessibility of many modern manufactured drugs [169]. Different research works have addressed the absorption and metabolism of tannins or their purified units. In accordance with their chemical nature, HT are more easily absorbed once they reach the small intestine since they are subjected to partial hydrolyzation by digestive acids. An experiment performed on rats analyzed the absorption of procyanidin dimers A1 [epicatechin-(2-O-7, 4–8)-catechin], A2 [epicatechin-(2-O-7, 4-8)-epicatechin], B2 [epicatechin-(4-8)-epicatechin], and other types and mixtures of procyanidins. All the three dimers were absorbed at the small intestine level and showed no conjugation or further chemical modifications (methylation), besides slight metabolization into monomeric epicatechin was observed. Among the dimers A1 and A2, they showed better absorption than B2, even though the presence of tetramers improved the absorption of B2 dimers. The monomeric epicatechin got partially methylated and totally conjugated. A-type trimers were not absorbed [170]. In another study developed with human adenocarcinoma stomach cell line (MKN-28), the transportation of procyanidins A2 and B2, (−)-epicatechin and (+)-catechin was analyzed. In concordance with the previous work, a transport of 23, 13 and 16% was determined for A2, (−)-epicatechin and (+)-catechin, respectively, showing a pH- and time-dependent transport pattern [171].

A review paper has evaluated the absorption, metabolism, distribution and excretion of (−)-epicatechin. As the previous study has pointed (–)-epicatechin is absorbed in the small intestine and can be detected in plasma at amounts under the μmol/L level one hour after its ingestion. Up to twelve structural-related (–)-epicatechin metabolites (SREMs) can be detected; however, the chemical profile is different between species. Indeed, in humans, the main SREMs are (−)-epicatechin-3′-sulfate and -glucuronide, while in rats are 3′-methyl-(−)-epicatechin and the -5-glucuronide form as well as the (−)-epicatechin-5-glucuronide. Later, flavan-3-ol units that reach the colon are transformed by microbiota gut into 5C-ring fission metabolites (5C-RFMs) 5-(hydroxyphenyl)-γ-valerolactones and 5-(hydroxyphenyl)–γ-hydroxyvaleric. These metabolites were quantified as 42% of the ingested (–)-epicatechin after nearly 6 h after its ingestion. The total (–)-epicatechin in urine and plasma can be quantified in terms of 5C-RFMs equivalents. Its presence in urine was determined at 95%, suggesting its high bioavailability. Similarly, a porcine model showed that acorns (*Quercus* sp.) ET were gradually fragmented to ellagic acid glucuronides and several urolithins. Subsequent decarboxylation, and dehydroxylation reactions loss of hydroxyl groups from ellagic acid to urolithins D, C, A and finally B decreased its antioxidant capacity by the lower hydroxyl groups but raised its lipophilicity. These urolithins were detected in bile and plasma, which indicates active circulation [172]. Urolithin A is also considered a biomarker of ET intake, as it is also detected in human plasma and urine. Administration of *R. idaeus* in healthy, and ileostomy patients showed the presence of urolithin A and B glucuronides in the urine of healthy subjects, while these were significantly lower in those with ileostomy [173]. This preeminent role of gut microbiota in improving the bioaccessibility of tannins in humans and animals has been addressed in other studies. One tested fermenting walnuts and punicalagin with human fecal bacteria, determining the production of urolithin A from ellagic acid. Results showed great variations among the subjects, with some bacterial samples displaying no detectable production, which supports the hypothesis that individual gut differences have a considerable effect on the bioaccessibility of molecules [174].

Thus, differences in the chemical profile of metabolites obtained from (–)-epicatechin depending on the studied species must be considered for extrapolating results between species. Moreover, it is necessary to establish appropriate concentrations, determine the adequate metabolites to experiment with, as well as the biomarkers that are going to be used for quantification purposes [175].

## 5. Traditional and Scientific Knowledge: Building Bridges

Considering records on the traditional use of selected plants and knowledge on their high levels of tannins, known to exert various bioactivities, a correlation could be suggested.

Some of the traditional uses with pharmacological applications of the genus *Acacia* indicated it as useful plants for treating gastrointestinal, respiratory or skin mild inflammatory processes, as well as having a narcotic effect useful to treat nervous system disorders [29,30]. Currently, it has been demonstrated that tannins present in *A. mearnsii* like robinetinidol-(4-β-8)-epigallocatechin 3-gallate was capable of restoring the oxidative damage induced in neuroblastoma cells [176]. Fisetinidol-derivatives from *A. mearnsii* also reduced the oxidative stress but also minimized the presence of inflammatory markers in macrophages, concordant effects of those from *A. nilotica* (provided anti-nociceptive, anti-inflammatory and antipyretic) tested in vivo [30,31,32].

Another plant with an important presence of tannins, *J. regia,* has also been applied as an anti-inflammatory (for rheumatism, hemorrhoids, mild skin inflammations) to reduce varicose veins or toothache [37,38]. These traditional uses can be related to the demonstrated capacity of EA as antioxidant, anti-inflammatory and antiatherogenic. EA, present in *J. regia* both free or as part of ETs, possesses the capacity of reducing the activity of lactate dehydrogenase, superoxide dismutase, catalase, and levels of hydroperoxides and oxidized glutathione (antioxidant capacity directly related with the anti-inflammatory) and avoids the cellular activity of serum creatine kinase-MB, VCAM-1 and ICAM-1 (antiatherogenic) [39,40,41].

Traditional uses of *Quercus* sp. with pharmacological aims have also been supported by scientific evidence. *Quercus* sp. was traditionally reported to be utilized for controlling diabetes, and, in fact, extracts obtained from *Quercus* sp. were demonstrated to inhibit α-glucosidase, a molecule used to evaluate antidiabetic properties in vitro [37,50]. Other traditional uses such as the treatment of mild inflammations may be connected with its demonstrated antioxidant properties that may reduce oxidative stress triggered by inflammatory cascades and reduce their impact [51].

The *Rubus* genus has a wide variety of traditional applications that cover the treatment of gastrointestinal, urinary, respiratory, eye and skin affections coursing inflammation, diabetes or atherosclerosis (was also used as anti-hemorrhagic [37,42]. Among them, *R. idaeus* was specifically evaluated as an inhibitor of platelet aggregation showing its capacity to modulate gene expression of proteins involved in the activation of platelets accompanied by a reduction in the oxidative status of the cell model [177]. These capacities are consistent with the reduction of the oxidative stress of hypertrophied adipocytes, where *R. idaeus* was also claimed to mobilize lipids [141]. These combined properties point to its potential use as a cardioprotective and key ingredient in the prevention of obesity. Antioxidant and anti-inflammatory general properties of *Rubus* are summed to the antidiabetic and gastroprotective ability demonstrated in vivo assays for *R. fruticosus* [124,178] or the healing capacity of *R. imperialis* [179]. These functions may be further supported by the potential antifungal and antibacterial capacities of plants belonging to this genus. *R. ulmifolius* was demonstrated to inhibit bacteria present in gastrointestinal, urinary or genital microbiota such as *Helicobacter pylori* [180], *Escherichia coli* or *Staphylococcus agalactiae* [181]. *R. ulmifolius* has been proven to possess antifungal activity against *C. albicans,* a common genital yeast [181]. It also has an antifungal effect against *Beauveria* sp. or *F. solani* involved in mycotic keratitis or endophthalmitis (eye care suggested by traditional uses), *M. canis*, *P. verrucosa* and *S. brevicaulis*, reported to cause skin and nail infections and lesions (treatment of mild skin affections was also traditionally reported) [182].

*Rhus* is a genus widely explored through scientific methods that concord with some of its traditional uses (gastrointestinal diseases such as dysentery, intestinal ulcers, rectal prolapse, or hemorrhoids, among others) [53]. The most studied species *R. coriaria* was demonstrated in vitro as capable of inhibiting TNF-α-induced inflammatory cascades [54] whereas, in vivo, it prevented necrotizing enterocolitis by reversing mild and focalized injuries [53], which can support its traditional use for gastrointestinal affections, including the treatment of intestinal ulcers, rectal prolapse or hemorrhoids. For these last properties, it is critical to know the accelerating healing capacity (prompted by both anti-inflammatory and antibacterial activity against *Staphylococcus aureus* or *Pseudomonas aeruginosa*) of this plant [183], while for its application against dysentery its role as anti-helminthic may provide some scientific basis [184].

Among the variability of the traditional uses of *Terminalia*, those related to gastrointestinal issues can be associated with the scientific finding. *T. chebula* was demonstrated to inhibit maltase, an enzyme present in the small intestine that catalyzes the maltose hydrolysis [102]. Moreover, *T. chebula* and *T. bellerica* also modulate genes involved in the storage and mobilization of lipids, glucose metabolism, morphogenesis and inflammatory response [185]. The capacity of improving the appetite of *T. chebula* may be indirectly related to its potential ability to ameliorate the expression of GABA receptors implied in relaxing mechanisms [186].

## 6. Discussion and Conclusions

Tannin-rich plants have been traditionally used in very different geographical areas, with autochthon species being used to treat a huge variety of affections. Some species showed several applications and are still used nowadays, which shows the great acceptance of bioactive molecules from these plants over the years. Moreover, it is estimated that 70% of the global population from underdeveloped countries do not have access to novel commercial drugs, being medicinal plants the only therapeutic solution.

This review offers an overview of the traditional uses of more than twenty-one genera of plants and the macroalgae *Sargassum* sp., all known to possess considerable amounts of tannins as a part of their tissues. Many species reported like *V. vinifera*, *J. regia*, *C. sativa* or *Sargassum* sp. are part of the diet of many countries without medicinal purposes, and its bioavailable low-polymerized tannins may be readily absorbed. Most typical applications in human bodies include the treatment of mild skin inflammations and gastrointestinal and respiratory affections. Therefore, this paper constitutes a useful tool to satisfy the global market, which demands natural phytomedicinal for diseases with difficult treatment like obesity, insomnia, anxiety, stress, constipation, etc. Plant applications in animals are mainly attributed to the anti-helminthic or antidiarrheal properties.

The chemical analysis of phytochemicals in traditionally used plants has been performed to figure out the composition of tannins and tannin-related molecules. Plants pose some common molecules like phenolic acids, GT, ET and polymeric flavonoids, and macroalgae contain PT, mainly fuhalols, fucols, ethols and carmalol derivatives. However, the variability in the chemical profile is high and mostly depended on the analyzed tissue, extraction procedure, selected solvent, geographical area and moment of collection, etc.

In summary, this review aimed to interconnect the knowledge collected along centuries in the traditional application of rich-tannins plants with scientific results to establish bridges between ethnology and science. We understand that ethnology compiles useful information achieved by trial and error of many generations, and now, based on works developed under the scientific methodology, this knowledge can be accepted or refuted. As previously exposed, some traditional uses have already been demonstrated by scientific data. Thus, those still not scientifically confirmed may represent an important source of information to develop innovative applications using tannins, natural ingredients with huge potential.

## Figures and Tables

**Table 1:** Traditional applications of plants containing tannins. Selection of species and tissues rich in tannins traditionally applied under diverse administration ways (admin.) for treating different affections or diseases and the potential mechanism of action of their biomolecules.

Traditional Use of Plants and Macroalgae Rich in Tannins				
Plant	Admin.	Treatment, Remedy, Uses	Mechanism of Action	Ref.
PLANTS				
***Acacia***				
*A. nilotica*	O, T	Gastrointestinal, respiratory, inflammatory, parasitic, neurological diseases, sexual disorders, skin issues, diabetes. Aphrodisiac, chemo-preventive, antimutagenic	Antioxidant, anti-inflammatory, anti-nociceptive, and antipyretic	[29,30,31,32,33]
*A. arabica*	O (G, S)	Used for sweetmeats (G) or roasted (S, India)		
*A. tortilis*	O, T	Gastrointestinal disorders in camelids, skin issues (edema, allergic dermatitis, wound/burns healing)	Antiparasitic and anti-inflammatory	[34]
***Betula***				
*B. pendula*	O (B in I/D)	Urinary, respiratory affections. Systematic diseases	Anti-viral	[35,36]
***Juglans***				
*J. regia*	O (N), T	Hemorrhoids, rheumatism, varicose veins, skin wounds, fever, cough, toothache, infecundity. Local analgesic. Hypercholesterolemic, antidiabetic, cardiotonic, vasodilator. Aromatizer. Antiparasitic	Anti-platelet, cardioprotective, antiatherogenic and anti-inflammatory	[37,38,39,40,41,42]
***Picea***				
*P. abies*	O (Sp/L/F/R/B)	Food ingredients or supplements (Sp, L, F, R). Bread-preparing flour or thicker in soups (B)	Antioxidant, antimicrobial, preservative	[43]
***Pistacia***				
*P. lentiscus*	O, T (St, FR-oil)	Improvement of gastrointestinal function. Infected wounds, scabies, bloat, constipation	Antiparasitic, anti-inflammatory	[44,45,46,47]
***Phyllanthus***				
*P. niruri*	O (L and FR)	Liver diseases (jaundice), urinary infections, inflammatory processes and malaria	Anti-inflammatory, antioxidant, hypoglycemic, hypolipidemic, hepatoprotective	[48,49]
***Quercus***				
*Quercus* sp.	O, T (R/S in D/FR)	Skin injuries (burn, boil wound). Respiratory affections (cold and flu). Diabetes	Antioxidant, antidiabetic	[37,42,50,51,52]
***Rhus***				
*Rhus* sp.	O	Gastrointestinal diseases (diarrhea, ulcers, hemorrhoids), dysentery, or stroke	Antimicrobial, anti-inflammatory, antiapoptotic, immunomodulatory, healing	[53,54]
***Schinopsis***				
*Schinopsis* sp.	O, T (I/D of L/B/Rs/FR/Br/C/W/S)	Anti-inflammatory, antimicrobial, antipyretic, astringent and cicatrizing. Respiration affections (cold, cough, asthma), stomachache, headache, dysentery or fractures	Antioxidant, antimicrobial, anthelmintic	[1,55,56,57,58,59,60]
***Smilax***				
*S. aspera*	O, T (D)	Urinary retention, antiseptic in cows, enhancing health state of rabbits, treatment of purulent vesicles	Antioxidant, anti-inflammatory, diuretic	[47,61]
***Umbilicus***				
*U. rupestris*	O, T (minced L)	Infected wounds, diarrhea, fever, intoxications, antiparasitic in hens	Anti-inflammatory, antiparasitic	[47,61,62]
***Urtica***				
*U. dioica*	O, T (I, direct application)	Arthritis, lumbago, rheumatism, muscular or limb paralysis. Rubefacient, blood circulation stimulant. Relief allergic rhinitis symptoms. Revitalizing. In animal promotes weight gain, growth and increases galactagogue production (ruminants)	Antioxidant, anti-inflammatory, antimicrobial, analgesic, anti-diabetic, antimutagenic. Emulsifier, gelling agent	[63,64,65,66,67,68]
***Vitis***				
*V. vinifera*	O (raw sp, vinegar)	Gastrointestinal diseases, headaches, and colds. Thirst-quenching, revitalizing and anti-inflammatory	Antioxidant, anti-obesity, anti-inflammatory	[42,69]
**Combination of plants**				
“Triphala”	Oral	Restorative, revitalizing, boosting of the immune system, treatment for chronic gastrointestinal diseases	-	[48,70]
**MACROALGAE**				
***Sargassum***				
*Sargassum* sp.	O, T	Nutritional value. Treatment for inflammations, goiter, dropsy, edema, dysuria, respiratory affections, angina pectoris, high blood pressure, skin diseases, neurosis, pregnancy-related depression and diabetes mellitus	Antioxidant, antibacterial, antiproliferative, anti-inflammatory. Gelling hydrocolloid, emulsifier	[71,72,73,74]
***Ecklonia***				
*E. cava*	Oral	Common food ingredient, attenuation of goiter, treatment for mammary hyperplasia and diuretic	Antioxidant, anti-inflammatory	[71,75,76]

Definitions: preparation modes: D: decoction; I: infusion; Pp: plant parts: B: bark; Br: branches; Bu: buds; C: cortex; F: flowers; FR: fruits; G: gum; J: jam; L: leaves; N: nuts; Rs: resin; R: roots; S: sap; Sp: sprouts; St: stems; W: wood.

**Table 2:** Rich-tannins species of plant and macroalgae with the main tannin and tannin-based compounds.

Genera	Species	Representative Tannins and Relevant Related Molecules	Ref.
*Acacia* sp.	*A. catechu*	**CT monomers, dimers and trimers**: trihydroxiflavan, profisetidin, gallocatechin, prorobinetidin, 3,5,7,4′-tetrahydroxyflavan	[110]
	*A. mearnsii*	**CT monomers, dimers and trimers**: fisetinidol, quercetin, myricetin, prodelphinidin and gallocatechin	[111,112]
		**Relevant dimers**:	
		robinetinidol-(4α-8)-gallocatechin	
		fisetinidol-(4α-8)-catechin and robinetinidol-(4α-8)-catechin	
		robinetinidol-(4α-8”)-robinetinidol (4′α-6”)-catechin	
	*A. nilotica*	**Phenolic acids** GA and EA	[30,113]
		**GT:** methyl gallate	
		**Polygalloyl units:**	
		ethyl gallate-1-galloyl-β-D-glucose, diGA and dicatechin	
		1,6-di-galloyl-β-D-glucose and gallocatechin-5-gallate	
		epigallocatechin-7-gallate and -5,7-digallate	
*Betula* sp.	*B. pendula*	**Phenolic acids**: glycosylated flavonoids and salicylates	[114,115]
		**CT**: oligomeric and polymeric flavan-3-ols	
		**Flavonoid-aglycones**: apigenin, luteolin, chrysoeriol derivatives	
*Castanea* sp.	*C. sativa*	**Phenolic acids:** EA	[101,116,117,118]
		**GT and ET:** chestanin, chesnatin, isochesnatin, chebulagic acid, pedunculagin, tellimagrandin I, castalagin/vescalagin, stachyurin or casuarinin, deoxyhexoside	
		**Other molecules:** cocciferin d2, castacrenin A-C isomers, trimethyl-ellagic acid hexoside, cretanin, methylvescalagin, vescavaloninic acid	
*Ecklonia* sp.	*E. cava*	**PT**: phloroglucinol, eckol, dieckol, 7-phloroeckol, 2,7-phloroglucinol-6,6-bieckol, phlorofucofuroeckol-A, pyrogallol-phloroglucinol-6,6-bieckol	[104,119]
*Juglans* sp.	*J. regia*	**HT:** pedunculagin, casuariin, valoneoyl, sanguisorboyl, tergalloyltellimagrandin I, praecoxin A, platycariin, glansrin C, alnusnin Bpterocarinin, breginin A and alienanin B, flavogallonic acid dilactone	[39]
*Lotus* sp.	*L. corniculatus*	**Tannin heteropolymers**: units of catechin/epicatechin and gallocatechin/epigallocatechin	[120]
*Picea* sp.	*P. abies*	**CT monomers**: catechin, epicatechin, gallocatechin, delphinidin	[121,122]
		**Tannin-related molecules**: myricetin; astringin, piceid, isorhapontin	
*Quercus* sp.	*Q. robur*	**Phenolic acids**: EA and GA	[50]
		**Ellagitannins:** castalagin, grandinin, castalin, vescalagin, vescalin	
		**Triterpenoid glycosides**: polygalloylquinic acid derivatives	
*Rhus* sp.	*R. coriaria*	**Phenolic acid:** GA	[54,123]
		**QUERG**	
		**CYANG derivatives:**	
		cyanidin-3-(2″galloyl)-galactoside and methyl delphinidin aglycone	
		7-methyl-delphinidin-3-(2″galloyl)-galactoside	
		7-methyl-cyanidin-3-(2″galloyl)-galactoside)	
		**Other bioactive compounds:**	
		galloylhexose, benzoic acid, galloylquinic acid and quinic acid	
		3,4, 5-trihydroxy-, 2-oxo-1,3-propanediyl ester	
		myricetin galloylhexoside, triGA	
*Rubus* sp.	*R. fruticosus*	**Phenolic acid:** GA	[124]
		**CYANG** and **vanillic acid**	
		**Flavonoids**: flavanone naringenin	
		**Anthocyanins** and **anthocyanidins:** malvidin-3-galactoside, cyanidin-3-galactoside and delphinidin-3-galactoside	
*Sapium* sp.	*S. baccatum,*	**Flavonoid quercetin 3-α-L-arabinopyranoside tannins**:	[125]
		methyl gallate, corilagin and tercatain	
		chebulagic acid and chebulinic acid	
*Sargassum* sp.	*S. fusiforme*	**PT:** fuhalols, fucols, ethols, carmalol derivatives	[73]
	*S. muticum*	**PT:** fuhalols (hydroxytrifuhalol B, hydroxypentafuhalol A, hydroxyheptafuhalol B and hydroxynonafuhalol A)	[126]
*Schinopsis* sp.	*S. lorentzii*	**GT**: TGG, PGG, FIS-catechin polymers and quinic acid-GA esters.	[59]
		**CT**: catechin-fisetinidol polymers	
	*S. balansae*	**CT**: fisetinidol and robinetinidol polymers	[127]
*Terminalia* sp.	*T. chebula*	**Phenolic acids:** EA, GA, chebulinic acid, chebulic acid.	[95,128]
		**GT**: TGG, methyl gallate	
		**ET:** chebulanin, methyl neo-chebulanin, corilagin, punicalagin, terflavin, flavogallonic acid, gallagic acid, methyl flavogallonate.	
		**Simple gallate esters:**	
		1,6-di-galloyl-β-ᴅ-glucose and 3,4,6-tri-galloyl-β-ᴅ-glucose	
		1,3,4,6-tetra-galloyl-β-ᴅ-glucose and 1,2,3,4,6-penta-galloyl-β-ᴅ-glucose	
*Vitis* sp.	*V. vinifera*	**PC, PD, galloylated-PC** and **flavan-3-ols**	[129,130]
		**Epicatechins**: epigallocatechin, epicatechin-3-gallate and procyanidins B, catechin-gallocatechin dimers and fisetinidin dimers	

Definitions: CAST: castalagin, CYANG: cyanidin-3-glucoside, EA: ellagic acid, ET: elagitannin, FIS: fisetinidin, GA: gallic acid, GT: gallotannin, PC: procyanidin, PD: prodelphinidin, PG: phloroglucinol; PGG: pentagalloylglucose, PoGG: polygalloylglucose PT: phlorotannins, QUERG: quercetin-3-glucoside TGG: trigalloylglucose, VES: vescalagin.

## Data Availability

Not applicable.

## Abbreviations

CoT	Complex tannin
CT	Condensed tannins
EA	Ellagic acid
ET	Ellagitannin
GA	Gallic acid
GT	Gallotannin
HHDP	Hexahydroxydiphenol
HT	Hydrolyzable tannins
NHTP	Nonahydroxytriphenoyl
PC	Procyanidin
PD	Prodelphinidin
PG	Phloroglucinol
TGG	Trigalloylglucose
PGG	Pentagalloylglucose
PT	Phlorotannins
GABA	Gamma-Aminobutyric acid
ICAM	Intercellular adhesion molecule
TNF-α	Tumor necrosis factor-α
VCAM	Vascular cell adhesion protein
